# Molecular Dispersion of Starch as a Crucial Parameter during Size-Exclusion Chromatography

**DOI:** 10.3390/foods9091204

**Published:** 2020-09-01

**Authors:** Artur Szwengiel, Piotr Kubiak

**Affiliations:** 1 Institute of Food Technology of Plant Origin, Faculty of Food Science and Nutrition, Poznań University of Life Sciences, Wojska Polskiego 31, 60–624 Poznań, Poland; 2 Department of Biotechnology and Food Microbiology, Poznań University of Life Sciences, 60–627 Poznań, Poland; piotr.kubiak@up.poznan.pl

**Keywords:** corn starch, potato starch, solubilization, size-exclusion chromatography (SEC)

## Abstract

Starch, α-polyglucan consisting of a large number of anhydroglucose units joined by α-1,4- and α-1,6-glycosidic bonds, seems to be characterized by a simple structure when compared to other natural polymers. Nevertheless, starches of various botanical origins have different physicochemical properties that are related to the differences in molecular and supramolecular structure of this polymer. In terms of the functional value of starch, the behavior of its macromolecules in solution is the most important result of its structural features. Extremely high molecular mass is the fundamental structural property of starch. Water, considered simply as a solvent for solubilization, does not provide molecular dispersion of starch without its degradation. The objectives of this study are to characterize the suitability of a new aqueous media (urea/NaOH) for enhancing the dispersion of native corn and potato starches and its effect on the consequent size-exclusion chromatography (SEC) analysis. The results were referred to other aqueous base solvents used for dispersing starch (NaOH and KOH). The samples were separated using SEC with triple detection and phosphate buffer (pH 8.0) with urea as the eluent. The characteristics of tested normal and waxy starches were compared. The results revealed that urea/NaOH did not degrade starch during the dispersion process. The recovery of starches, however, was not higher than 42%. These results prove that while the urea/NaOH solvent allows to obtain cold-water-soluble starch, the degree of disintegration of the intramolecular interactions of amylopectin chains is still insufficient.

## 1. Introduction

Starch is one of the most useful polymers in the food and non-food applications (paper, chemistry, fermentation, material and pharmaceutical industries). It is a biopolymer that is biodegradable and renewable [1]. It is also considered as a raw material for the production of green materials that could substitute synthetic polymers [2]. It is widely used as a thickening, stabilizing, and gelling component in the food industry. Low price and ease of obtaining starch are the main factors that encourage producers to use it on a large scale [3]. Edible plants, such as corn, potato, wheat, barley, cassava, rice, and sweet potato, are abundant sources of starch. [4] Commercially available starch is obtained mainly from corn [5] but potato starch is also widely used in a variety of products [6].

Generally, starch granules consist of amylose (AM) and amylopectin (AP) as well as minor components–proteins, lipids, and minerals [7]. AP has a high molecular weight and is highly branched. It contains anhydroglucose chains linked by (1→4)-α glycosidic linkages. Branches are formed by (1→6)-α linkages [8,9]. The structure of AM is predominately linear with α (1→4)-linkages and rare branches. The molecular mass of amylose (approximately 10^6^ Da) is much lower than amylopectin which is known as one of the largest biopolymers (approximately 10^8^ Da) [10]. The structural variability and the ratio of AM to AP is strongly dependent on the botanical origin of starch [11]. Generally, AM fills spaces within the AP matrix. AP is branched with 4–5% α-1,6 linkages, short AP chains are oriented within clusters that are connected by longer chains. Long linear chain segments of AP form double helices which pack into ordered lamellar arrays [12].

Starch is classified into four crystalline types (A, B, C, and V) according to X-ray diffraction (XRD). The A-type crystalline pattern is typical for most cereal starches, such as corn, wheat, and rice starch. Tuber starches (potato starch) with their high amylose starch content belong to B-type crystalline type. The combination of A- and B-type crystalline pattern is typical for legume starches, classified as type C. A V-type crystalline pattern is observed when amylose complexes with fatty acids or alcohols [13]. In general, regular starches contain about 70–80% AP and 20–30% AM. High-AM starches contain more than 40% AM, and waxy starches contain more than 90% AP [11]. Granule-bound starch synthase I (GBSSI) enzyme is responsible for the biosynthesis of amylose. Cereal waxy starches and amylose-free starches in potato are a result of either low or no expression of the GBSS gene [14,15]. The molecular structure and the ratio of AM/AP in starch granule have influence on the gelatinization temperature, solubility, viscosity, gelation, and retrogradation properties which are in turn reflected in the texture and stability of starch-based products [16]. Swollen granules of waxy-starches are highly susceptible to mechanical breakdown and solubilized faster than starches with a higher content of AM. In the latter case, extensive granule swelling is inhibited. High water retention capacity (1.2–1.5×) and higher viscosity of pastes (up to 40% for corn) were observed for waxy starches [17]. Waxy starches are also more resistant to retrogradation, a crystallization process which occurs during ageing of gels of normal starches within a day, and this advantage is utilized in polymer and food applications [8]. It was also reported that amylopectin of normal starch has a lower molecular weight than that of waxy starch counterpart. It is probably attributed to the carbon flux which is not directed exclusively to amylopectin in the biosynthesis of normal starch [18]. Extra-long branch chains of amylopectin are found in normal starches while amylopectins of waxy starches have more branch-chains. This results in less densely packed molecules of amylopectin in normal starches compared to amylopectin of waxy starches [19].

Properties of starch are also determined by other compounds. Root or tuber starches contain little or no lipids while significant amounts of lipids are complexed with amylose in amylose-containing cereal starches. Cereal and root (tapioca) contain lower phosphate monoester content than tuber starches (canna, potato) [20]. Interaction between lipids and amylose can occur upon starch gelatinization, especially in cereal grains where lipids occur in the native granules of starch or surround the granules [21]. Lipid-amylose complexes have impact on the formation and content of resistant starch of type V, swelling capacity in water, pasting characteristics, solubility, and gel texture [22]. Phosphate groups have impact on the rheological properties of starch. Their presence results in increased viscosity and clearness of gels which is advantageous for many industrial applications [23]. 

It is often required that the starch withstands high temperatures, low pH, and high shear forces [6]. Therefore starches are modified physically (pre-gelatinization, heat-moisture treatment, annealing, high pressure treatment) and chemically (acid hydrolysis, substitution, cross-linking, oxidation) since native starches usually do not meet industrial needs.

It is a huge challenge that is driven by high demand to destroy the molecular hydrogen bonds in starch in order to extend the range of applications of and enhance the processability of this biopolymer [2,24]. Hydrogen bonding and areas of crystallinity render granules of starch insoluble in water at room temperature. Highly swollen but insoluble granules and granule fragments can be separated from aqueous solution after heating although an increase of solubility in water is observed as temperatures approach 95–100 °C [25]. Swollen hydrated forms, known as ghost structures, are typically found after gelatinization in water under common heating conditions (below 100 °C) [26]. Dimethyl sulfoxide (DMSO) is one of the known organic solvents which makes it possible to plasticize and disrupt hydrogen bonds formed between polysaccharide chains [27]. Alkaline solutions (NaOH, KOH) are also commonly used to disperse starch [28]. Some application of zinc chloride aqueous solution [29], molten imidazole [27], 4-methylmorpholine 4-oxide (NMMO) [30], and NaOH/urea [31,32] were also proposed recently. Ionic liquids, promising “green solvents,” can be used for the processing of polysaccharides, however they have a tendency to cause starch degradation and their potential toxicity limits their applicability in starch-based foods [2].

There is no general agreement as to the best approach to the characterization of starch which is a huge and highly branched molecule. However, it is very important to determine not only the average molecular mass of starch but also its distribution. Samples with the same molecular mass but with very different distribution could manifest different rheological properties. The knowledge of the average molecular mass and radius of gyration of starch is important for its industrial application, for example: (i) Beverage thickeners—too high molecular mass makes the product too viscous with a chalky or slimy texture; (ii) resistant starch-control of digestive properties and retrogradation requires molecular mass in the correct range; (iii) enteral nutrition solutions–excessive molecular mass reduces energy supply and causes clogging of the tubing [33].

The biggest problem in determining the molecular mass of starch is the necessity to fully disperse the sample (disperse as separate molecules) without its degradation [33]. However, it was noted that even under minimal shear conditions, such as gentle agitation, the amylopectin molecules are very susceptible to shear degradation [34].

Whole starch molecules are usually separated using common techniques of size-exclusion chromatography (SEC), field-flow fractionation (FFF), analytical ultracentrifugation (AUC), and hydrodynamic chromatography (HDC) [35]. SEC can be performed in an aqueous medium and then is denoted as gel filtration chromatography (GFC), whereas gel permeation chromatography (GPC) is a term used when an organic solvent is used for elution. Generally, the term SEC is preferred since it is all-inclusive and more aptly descriptive [36]. SEC and GPC are often used as synonyms. The structural analysis of starch in aqueous media is difficult because starch chains have limited stability in neutral aqueous solutions which results in inaccuracy of the determination of molar masses which stems from incomplete dispersion or chain aggregation [37].

Unfortunately, dispersing starch in DMSO is also not straightforward, mainly because the required conditions (time, temperature, stirring) are not universal to all starches. Namely, amylose-rich corn starch is easier to disperse in DMSO than waxy starch. Excessive mechanical stirring, high temperature, excessive boiling (2 h or longer in water bath at 100 °C), autoclaving (121 °C, 15 min) can lead to an increased size of the apparent amylose peak as a consequence of amylopectin degradation [34]. However, this is not the only problem of using organic SEC with DMSO. The determination of the weight average molecular mass of starch (*M_w_*) with static light scattering (SLS) is limited since light scattering intensity is dependent on the increment in the refractive index (dn/dc) [38]. Comparison of dn/dc for starch in DMSO (0.066 mL/g) or DMSO/H_2_O (90/10, dn/dc = 0.074 mL/g) [39] with dn/dc value in aqueous SEC (0.160 mL/g for 0.1 M sodium nitrate in water with 0.02% sodium azide) makes it clear that detector response is significantly decreased in DMSO.

Summing up, the dispersion of starch can be obtained by the formation of hydrogen bodings with a protonic solvent (water) or dipolar aprotic solvent (DMSO), or by hydration of the ionized hydroxyl groups of starch in presence of a base (NaOH) [31]. However, there is still a need for cheap and environmentally friendly solvents that guarantee real dispersion of starch where each molecule is surrounded by the solvent. Recently, urea/NaOH aqueous solutions were proposed [31,32,40] since urea is known as a good plasticizer for starch [41,42] and NaOH shows good dissolving capacity toward the polymer [43].

The aim of this study is to determine the suitability of urea/NaOH aqueous media for enhancing the dispersion of starch for the purpose of SEC analysis. Normal and waxy starches from corn and potato were tested as the most popular examples for A-and B-type crystalline pattern. The samples were neutralized immediately after the dispersion process using phosphoric acid, then diluted directly in the alkaline mobile phase (phosphate buffer (pH 8.0) with urea). The idea was to verify the possibility to characterize starch in an aqueous solvent which is its natural plasticizer and which enables to reflect the conditions encountered in food matrices. The obtained results were then compared with the effectiveness of dispersion in aqueous solutions of NaOH and KOH.

## 2. Materials and Methods

### 2.1. Starch Samples and Chemicals

The study was performed with commercial native potato starch, Superior Standard (PPZ Trzemeszno, Poland), waxy potato starch, Eliane (Avebe, Groningen, Netherlands), corn starch (donated by the Department of Food Concentrates, Institute of Agricultural and Food Biotechnology, Poznań, Poland), waxy corn starch (with trace amounts of amylose; Sigma, S-9679). Pullulan and amylose from potato standards were purchased from Shodex (Tokyo, Japan) and Sigma–Aldrich Chemie GmbH (Munich, Germany), respectively. Urea, Na_2_HPO_4_, NaH_2_PO_4_, NaOH, KOH, H_3_PO_4_, sucrose were obtained from Avantor Performance Materials Poland S.A. (Gliwice, Poland).

### 2.2. Determination of Dn/Dc in a New Eluent

An Abbe refractometer was used to determine dn/dc for sucrose dissolved in 50 mM phosphate buffer (pH 8.0) with 100 mM urea at concentrations in the range from 0 to 0.3 g/mL. Measurements were performed at the temperature of 50 °C. Methodology was adapted form Behrens et al. [44]. The dn/dc of pullulan standard (11.3 kDa; 0.8–3.1 mg/mL) and amylose from potato (0.3–1.2 mg/mL) in the same solvent were determined using SEC (Malvern, TX, USA) equipped with a conventional dual cell refractometer calibrated with sucrose solution using dn/dc determined previously with the Abbe refractometer. The calculations were performed using OmiSEC 4.7 software (Malvern, TX, USA). The accuracy of calculated dn/dc for pullulan was verified by pullulan standards with molar masses in the range of 6.15–2460 kDa. The justification of predefining dn/dc in a new aqueous solvent using a sucrose solution is described in detail in the Section 3.

### 2.3. Alkaline Pasting and Dispersion of Starch

Starch samples of 30 mg were allowed to disperse in 4.5 mL of urea (2.3 M)/NaOH (1 M) aqueous solution [31] at 0 °C for 24 h and then were neutralized with 0.5 M H_3_PO_4_ and diluted with the mobile phase used for SEC separation. Results for corn starch were compared with other alkaline dispersion methods which employed 0.5 or 1 M NaOH [45] or 2 M KOH [46,47] at 65 °C and stirring with a magnetic bar at 400 rpm. Briefly, 30 mg of starch were wetted by 50 µL of ethanol to prevent lumping. The alkali solutions (65 °C) were added into stirred sample placed in a water bath at 65 °C. Three procedures were applied: (i) 0.5 mL 1 M NaOH was added to a sample of corn starch and stirred gently for 2 min after which 4 mL of water were introduced and the dispersion was continued for 1 h; (ii) 0.5 mL 0.5 M NaOH was added to a sample of corn starch and stirred gently for 2 min after which 4 mL of water were introduced and the dispersion was continued for 1 h; (iii) 4.5 mL 2 N KOH was added to starch sample, dispersion time was 1 h as previously. After the dispersion with NaOH/KOH, the samples (variants (i)–(iii)) were neutralized with 0.5 M H_3_PO_4_ and diluted with the mobile phase used for SEC. The final concentration of all samples was 1.2 mg/mL. The samples were clarified using 5 µm PTFE filters (Merck Millipore, Ireland). The injection volume was 50 µL. The experiments were conducted in triplicate.

### 2.4. Molecular Characterization Using SEC with Triple Detection

SEC equipment (Malvern, TX, USA) with triple detection (Viscotek 305 TDA) was used for the separation of samples. A conventional dual cell refractometer (RI), viscometer (Vis), and light scattering (low-angle light scattering, LALS and right-angle light scattering RALS) detectors were employed. Aqueous SEC analysis was performed using two aqueous SEC columns (Shodex OHpak SB-800HQ series) with a guard SB-G type column (Showa Denko, Tokyo, Japan). Total of 50 mM phosphate buffer (pH 8.0) with 100 mM urea was used as the eluent at a flow rate of 0.4 mL/min. The detectors and column were in the same oven operated at 50 °C. Calibration was performed with a pullulan standard (11.3 kDa). The refractive index of the solvent was 1.334. The calculations were performed using OmiSEC 4.7 software (Malvern, TX, USA).

### 2.5. Calculations, Statistical Analysis

RI signal (RI_area_) is described by the following equation: RI_area_ = k′ × (dn/dc) × m_inj_ [48], where: k′ is the instrument constant of the RI detector (*k*/*n*_0_; the refractometer response constant, *k*, the refractive index of the solvent, *n*_0_), m_inj_ is the total injected mass calculated from the injected concentration and the injection volume (m_inj_ = c_inj_ × V_inj_). The *M_w_* was calculated from the Rayleigh equation limited for low scattering angles (*Kc*/∆*R_θ_ =* (1/M_w_ *+* 2*A*_2_c)1/*P_θ_*,) where: *c* is the concentration of the polymer; ∆*R_θ_* the Rayleigh ratio (defined as the amount of light scattered by the analyte solution in excess of that scattered by the solvent at a given angle *θ*), *A*_2_ the second viral coefficient (in the limit of zero angle and near-infinite dilution this coefficient can be neglected); *K* the optical constant *K =* (4π^2^*n*_0_*^2^*/N_A_*λ*^4^)(dn/dc)^2^; *n*_0_ the solvent refractive index; *λ* the laser wavelength; and N_A_ is Avogadro’s number), *P_θ_* is a form factor related to the size of the molecule and the angle at which the scattering is determined, at *θ* = 0°, *P_θ_* = 1 [36,49,50]. LALS and RALS signals were used to calculate the radius of gyration *R_g_* because *P_θ_* can be determined for samples that scatter anisotropically. This in turn can be used to estimate a value of *R_g_* assuming a structural model such as a random coil or a hard sphere [36]. *R_g_* is the distance from the center of mass of a body at which the whole mass could be concentrated without changing its moment of rotational inertia about an axis through the center of mass [51]. The viscometric radius (*R_η_*) was calculated as the radius of a homogeneous sphere according to the formula *R_η_ =* ((3IV*M_w_*)/(10πN_A_))^1/3^, where: *IV* is the intrinsic viscosity, *M_w_* the molecular mass, N_A_ Avogadro’s number [50]. One-way ANOVA and Tukey test was used to test the significance of differences at alfa = 0.05. Statistical analysis was performed using Statistica version 10, StatSoft Inc. (Tulsa, OK, USA).

## 3. Results and Discussion

### 3.1. Determination of Dn/Dc Using a Calibrated RI Detector

**Figure 1:**
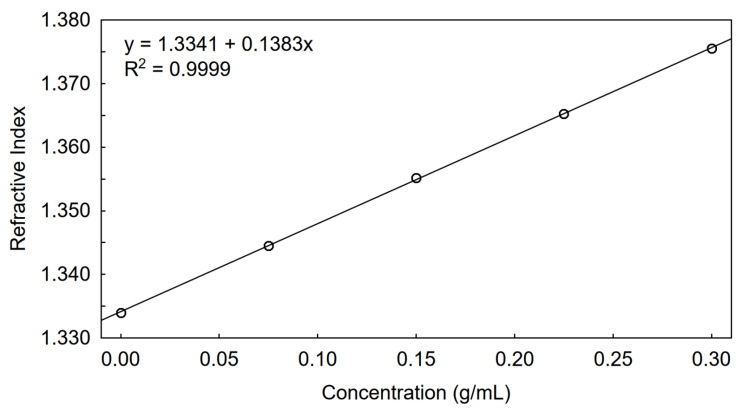
Determination of dn/dc for sucrose in 50 mM phosphate buffer (pH 8.0) with 100 mM urea using Abbe refractometer.

Calibration of the RI detector should be performed using the same solvent that is applied during SEC. Therefore, a reference sample with a known dn/dc in this solvent is required. Unfortunately, reference values for dn/dc cannot always be found in the literature, and an Abbe refractometer is usually not sensitive enough to determine dn/dc precisely in a low concentration range. It seems justified to use sucrose for the calibration of the RI detector since it dissolves in a wide range of concentrations in aqueous eluents and gives the opportunity to determine dn/dc precisely with an Abbe refractometer. The dn/dc determined for sucrose dissolved in the tested solvent is presented in Figure 1, the estimated dn/dc value in 50 mM phosphate buffer (pH 8.0) with 100 mM urea was 0.138 (mL/g) whereas the refractive index of the solvent n_0_ was 1.334. The calibration of the RI detector was done in the next step; the refractometer response constant (k) was 1.05689 × 10^3^.

**Figure 2:**
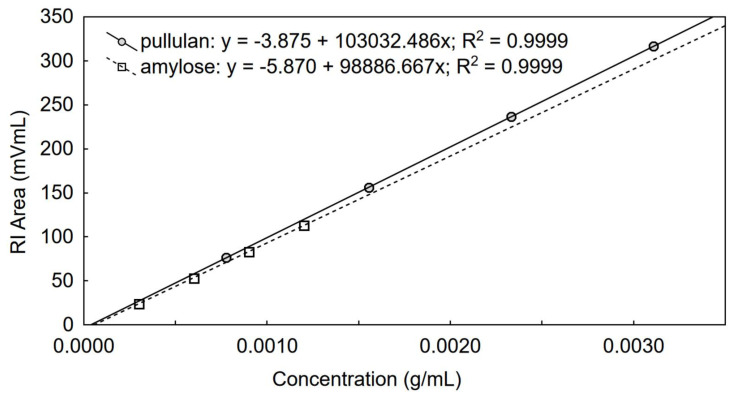
Chromatographic RI data used for the determination of dn/dc of pullulan and amylose from potato in 50 mM phosphate buffer (pH 8.0) with 100 mM urea, V_inj_ = 100 µL.

Since different polymers have unique RI responses it is necessary to precisely determine dn/dc for the tested types of polymers. The dn/dc parameter depends on the solvent, the temperature, the wavelength of light, the average molar mass (especially if *M_n_* is less than 10^3^ Da), the chemical structure of the polymer. Chain branching, however, has no effect on dn/dc [52]. Using the parameters estimated for sucrose and the refractometer response constant it was possible to determine dn/dc for the standards of pullulan and amylose from potato for the tested polymer solvent pairs using the RI detector. The results presented in Figure 2 gave the opportunity to calculate dn/dc as dn/dc = slope/(*k*/*n*_0_)/V_inj_. The calculated dn/dc values for pullulan and amylose from potato were 0.129 and 0.124 (mL/g), respectively.

Finally, the response constants and offsets (relative to the RI signal, mL) for other detectors (LS, Vis) were calculated with the pullulan standard (11.3 kDa). The recovery and accuracy of molecular mass determination were calculated for standards of pullulan (6.15–2460 kDa). The results (Table 1) showed that the recovery of pullulan standards was 99.99 ± 1.83%. The difference between the molecular mass declared by the producer and determined experimentally was not higher than 4.4%. The high recovery and low error of molar mass determination prove that the use of sucrose to predetermine the instrument constant of the RI detector can give accurate results. The procedure proposed here is simple, however, it is an unusual approach that gives the opportunity to determine the instrument constant using a new aqueous solvent. It is possible to directly determine dn/dc of the pullulan standard in a new solvent using an Abbe refractometer but this requires the use of a very sensitive refractometer or a standard, which is usually a limited resource, in a large amount.

### 3.2. SEC of Samples Dispersed in NaOH/Urea

**Figure 3:**
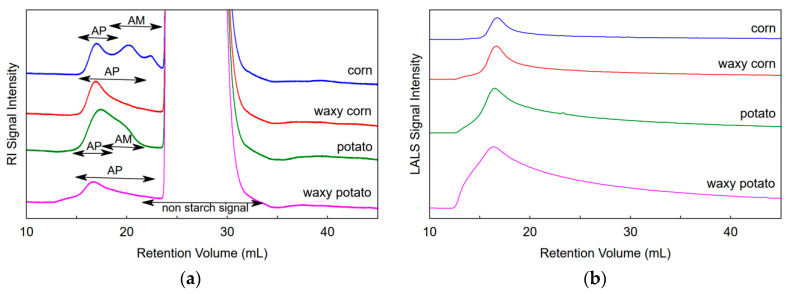

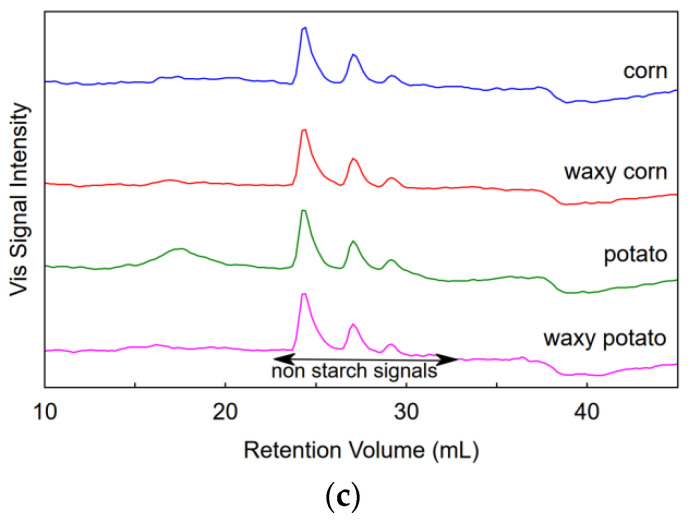
SEC chromatograms of corn, waxy corn, potato, waxy potato starch; (**a**) signals of the refractive index (RI) detector, (**b**) four-capillary viscometer (Vis) detector, (**c**) low-angle light scattering (LALS); the samples were dispersed in aqueous solution of urea/NaOH at 0 °C for 24 h and eluted with 50 mM phosphate buffer (pH 8.0) with 100 mM urea. The arrows indicate amylose (AM) and amylopectin (AP) ranges; the possible traces of amylose in waxy starches were neglected.

Following the dispersion in NaOH/urea at 0 °C, the starch samples were diluted and separated using SEC. Signals recorded with RI, Vis, and LALS detectors are presented in Figure 3. The starch molecules were eluted in retention volume ranging from 12.7 to 23.3 mL, Figure 3a. A trimodal peak distribution (RI signal) was observed for corn starch sample. The first peak (15–18.7 mL) corresponded to amylopectin, the second and third peaks—to amylose. The RI chromatogram of waxy corn starch contained a single peak (15–22.5 mL) with a noticeable tailing toward higher retention volumes. The profile of potato starch did not show a pronounced valley between amylopectin and amylose peaks recorded in the range of 15–22 mL. The waxy potato starch showed a pattern similar to waxy corn starch. Tailing of right side of the peak was observed together with a weak signal indicating large hydrodynamic volume recorded in range of retention volume from 12.7 to 15.4 mL. Intensive, non-starch signals were also observed since the samples were not precipitated after dispersion but neutralized and diluted directly. Thus, the excess of urea and phosphorus salts was recorded (23.4–34 mL). The samples diluted in the eluent (pH 8.0) did not precipitate in vials for 3 days and their SEC profiles did not change during 24 h. This is especially important in the analysis of a batch of samples. 

Light scattering (LS) signals (Figure 3b) were proportional to the molecular mass and concentration of the polymer. Other factors were constant (dn/dc and calibration factor of detector). It was observed that the LALS signal did not reach the level of the baseline from before the injection after 23.3 mL retention volume. This suggests that some part of sample gradually eluted from the column with a delay. It was especially apparent for waxy samples. Comparing the LS signals, it was noted that waxy potato starch consisted of molecules of the largest size (intensive LS signals and low RI signal simultaneously).

Surprisingly, the Vis detector (Figure 3) gave only a weak signal for potato starch. It is a huge disadvantage because without the Vis signal it was not possible to determine the viscosimetric radius (R_η_), intrinsic viscosity, parameters of Mark–Houwink (M–H) parameters, and M–H plot. Low polymer concentration or low polymer recovery, wide range of sample elution, incompatibility of solvent are the possible reasons for the weak signal of the Vis detector. Comparison of the signals for pullulan P2500 (elution range 15–20 mL) and potato starch with the same load on the column showed that the RI signal for pullulan (recovery close to 100%, dn/dc = 0.129 mL/g) was about 2.8 times lower than the signal for potato starch (recovery 42%, dn/dc = 0.124 mL/g). It proves that the main reasons for the weak signals from the Vis detector for the starch samples were mainly low recovery and a broad elution range (Table 2).

### 3.3. SEC of Samples Dispersed in NaOH or KOH

**Figure 4:**
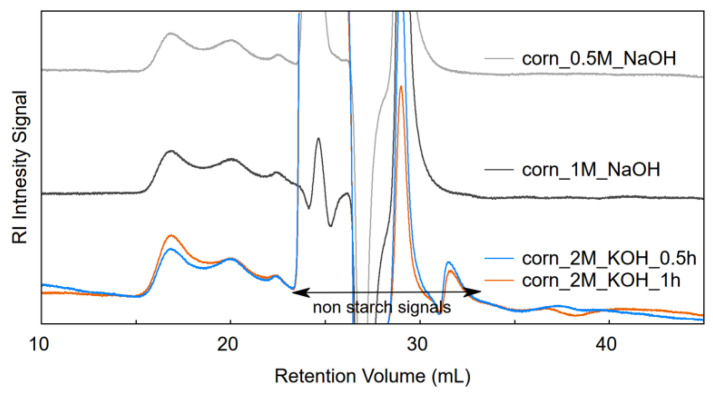
SEC chromatograms of corn starch samples dispersed in aqueous solution NaOH or KOH at 65 °C and eluted with 50 mM phosphate buffer (pH 8.0) with 100 mM urea.

Additional experiments were conducted to compare the effectiveness of dispersion of starch in urea (2.3 M)/NaOH (1 M) aqueous solution at 0 °C with other methods where base conditions are applied, i.e., using 0.5/1.0 M NaOH or 2 M KOH at 65 °C. Corn starch was used for this purpose. RI chromatograms of corn starch are presented in Figure 4, the SEC profiles were analogical to the ones presented previously for corn starch sample dispersed in urea/NaOH. The intensity of the amylopectin band was similar to the amylose band which suggests that amylopectin was not dispersed fully. Chromatograms where the sample was dispersed in 2 M KOH for 0.5 and 1 h, respectively, provide evidence that the low recovery of the samples was related to low dispersion of amylopectin (Figure 4). A more intensive signal for amylopectin (15–18.7 mL) was observed when a longer dispersion time was applied. At the same time the amylose signal remained unchanged. Dispersion of the samples in alkaline conditions with gentle stirring did not result in the destruction of amylopectin as indicated by the calculated content of amylose which oscillated within the range of 20–21%. The determined content of amylose in the tested sample was consistent with earlier determinations performed using the amylose/amylopectin test kit (Megazyme Ltd., Wicklow, Ireland) based on a Con A precipitation procedure [53] which proves a 100% amylose recovery.

### 3.4. Molecular Mass, Size, and Recovery of Starch Samples

Parameters calculated for the tested starches dispersed in urea/NaOH, 0.5/1.0 M NaOH, or 2 M KOH are presented in Table 2. The recovery of potato amylose was close to 100% and the determined weight average molar mass (*M_w_*) was typical for this fraction as determined previously [10]. The highest molecular masses and gyration radiuses (*R_g_*) were calculated for potato and waxy potato starches. Nonetheless, the recovery of waxy potato starch (21%) was significantly lower than the recovery of potato starch (42%) since the latter contains amylose which disperses fully in urea/NaOH solvent. Analogical difference was noted for corn and waxy corn starches. These observations provide evidence that the aqueous urea/NaOH solvent does not allow to properly disperse amylopectin. The highest recovery (58%) was obtained with 2.0 M KOH and corn starch in which case also the molar mass was determined to be higher compared to the respective sample dispersed in urea/NaOH but lower compared to corn starch dispersed in 1 M NaOH. Nonetheless, it is not possible to conclude that urea/NaOH solvent degrades amylopectin of corn starch more than NaOH or KOH. With such small recovery values it is possible that each of the applied solvents disperses a slightly different fraction of the sample.

## 4. Discussion

The nutritional and industrial importance of starch is unquestionable but its application requires knowledge of its structure and molecular size distribution. SEC is the most common technique used to characterize this distribution [54]. However, a uniform method of dispersion of starch for the purpose of SEC has not been established yet. Generally, water, dimethyl sulfoxide (DMSO), and N, N-dimethyloacetamide supplemented with LiCl are examples of systems in which starch undergoes dispersion but the conformation of chains and their behavior depend on the type of the solvent used [55]. Common food environments are aqueous but amylose retrogrades in water quickly and forms particles of uniform size [56]. The coils of amylopectin create a network in water that results from the entanglement of the side branches. [55]. Amylose predominantly takes the conformation of a helix in DMSO which transits into a loose helix and then into a random coil with an increasing water content [57]. Amylopectin demonstrates a compact spherical conformation in DMSO [58]. It can thus be deduced that the characterization of starch should be performed in an aqueous solvent as its structure under such conditions reflects its nature in food systems much closer than the structure it takes in an organic solvent. Performing SEC analysis with the use of an aqueous solvent is also advantageous in terms of signal-to-noise ratio since the dn/dc values are high compared to the small values that cause problems during analyses performed with DMSO [35]. Therefore, a solvent was proposed, a 50 mM phosphate buffer (pH 8.0) with 100 mM urea in case of which the dn/dc value was 0.124 for starch. The idea was to dilute starch after dispersion in the aqueous urea/NaOH solvent would be non-degradative and retrogradation-limiting. Aqueous media with pH in the range of 4–10 are considered non-degradative toward starch [59]. Increased pH values were also found to diminish the retrogradation of starch [60,61]. This corresponds to our observations as no precipitation of sample was observed prior to SEC analysis at pH 8.0. The dispersions of starch samples were stable for 3 days at 22 °C.

The proposed SEC conditions yielded repeatable SEC separations. The determined molar masses and R_g_ values were consistent throughout repeated injections performed over 24 h. Nevertheless, the weak signals recorded by the viscometric detector did enable to calculate the viscosimetric radius and intrinsic viscosity or apply a universal calibration validated for a number of branched polymers, including starch [62]. It was reported that urea and NaOH are good plasticization and dissolving factors for corn starch, respectively, and that the molar mass determined using an Ubbelohde viscometer and Mark-Houwink relation was 1.53 × 10^7^ Da [31]. A similar result was obtained in this study (2.13 × 10^7^ Da). Despite the fact that the solubility of corn starch in urea (2.3 M)/NaOH (1 M) was found to be 99% up to a concentration of 1% and pose no serious degradation problems [62], it was not possible to obtain a high recovery during SEC. However, degradation of amylopectin during dispersion was not observed as the calculated amylose concentration was typical for corn starch [53]. Autoclaving, microwave heating, homogenization provide better SEC recovery but result in degradation of starch molecules. The recovery of corn starch dispersed in an aqueous NaOH solution was found to be <50% (elution using aqueous NaNO_3_) [63]. When DMSO/water solution (90:10, *v*/*v*) was used to obtain starch dispersion prior to aqueous SEC the recovery was also not improved (41% recovery for corn starch) [53]. Dispersion of potato starch for 2 h in 1 M alkali aqueous solution at 25 °C yielded recovery rates of 85% during organic (DMSO) SEC [46].

It was also reported that 20–30% of large amylopectin molecules may not pass the SEC column and the injection membrane filter (3.0 µm) [63]. The samples in our experiments were therefore filtered using membranes of 5.0 µm porosity and the post column filter was removed from SEC equipment. The flow rate during SEC analysis did not cause degradation of amylopectin of corn and potato starch but tailing of peaks of the analyzed waxy starches was recorded. It is possible that a slight shear degradation took place during SEC separation since extensive shear scission that occurs in the column was suggested as the cause behind changes in the apparent size distribution of the amylopectin region [54]. A recent report shows that a 10-minute treatment of corn starch with urea and NaOH in higher concentrations (3.5 M and 2 M, respectively) and gentle stirring at ambient temperature results in obtaining granular cold-water-soluble starch. Nonetheless, it was accompanied by a slight increase in the amylose content due to the degradation of amylopectin [32].

## 5. Conclusions

The main problem that affects the analysis of the structure of starch using SEC is the poor solubility of this biopolymer in aqueous solvents. For this reason, DMSO is usually used as a solvent but its physical properties limit the sensitivity of RI and LS detectors. Moreover, utilization of this organic solvent does not allow to reflect the conditions in which starch is dispersed in the water phase of food. This justifies the investigation into the possibility of applying new aqueous solvents for SEC analysis of starches intended for food applications. The conducted experiments support previous conclusions that urea and NaOH are good plasticization and dissolving factors, respectively. However, the analysis of SEC data proves that urea/NaOH does not show as efficient dispersion of amylopectin as DMSO. At the same time the application of this aqueous solvent does not lead to the degradation of the sample which was also reported when aqueous solutions of NaOH were used. 

## Figures and Tables

**Table 1:** Molar masses and hydrodynamic parameters of pullulan standards (P5–P2500, Shodex, Japan) estimated by SEC method with triple detection after dispersion in 50 mM phosphate buffer (pH 8.0) with 100 mM urea; the values of the coefficient of variation (%) are given in brackets.

Standard	*M_n_*^1^ (Da)	*M_w_*^1^ (Da)	*M_w_* Expected ^2^ (Da)	*M_w_* Error ^2^ (%)	IV ^3^ (dL/g)	*R_η_*^4^ (nm)	*R_g_*^5^ (nm)	Recovery (%)
P5	5.79 × 10^3^ (1.8)	6.15 × 10^3^ (1.1)	6.00 × 10^3^	2.5	0.07 (0.4)	2 (1.0)	n/c	99.4 (0.3)
P10	9.86 × 10^3^ (0.92)	1.03 × 10^4^ (0.46)	1.00 × 10^4^	3.3	0.10 (12.2)	3 (5.5)	n/c	101.3 (0.1)
P20	2.14 × 10^4^ (0.95)	2.17 × 10^4^ (1.11)	2.17 × 10^4^	0.2	0.15 (0.9)	4 (2.5)	n/c	99.9 (1.1)
P50	4.67 × 10^4^ (1.64)	4.94 × 10^4^ (1.34)	4.88 × 10^4^	1.2	0.27 (0.0)	6 (0.7)	n/c	101.0 (0.8)
P200	1.95 × 10^5^ (2.54)	2.19 × 10^5^ (1.23)	2.10 × 10^5^	4.4	0.58 (4.6)	13 (1.1)	n/c	103.2 (1.2)
P400	3.44 × 10^5^ (0.94)	3.68 × 10^5^ (1.08)	3.66 × 10^5^	0.6	1.01 (10.1)	18 (5.1)	27 (5.2)	99.7 (1.2)
P800	7.18 × 10^5^ (2.73)	8.08 × 10^5^ (1.23)	8.05 × 10^5^	0.3	1.66 (5.8)	28 (2.3)	40 (6.4)	100.0 (1.9)
P1300	1.18 × 106 (3.91)	1.35 × 10^6^ (2.25)	1.33 × 10^6^	1.6	2.47 (2.8)	37 (3.6)	54 (4.4)	97.8 (1.3)
P2500	1.78 × 10^6^ (10.35)	2.46 × 10^6^ (2.33)	2.56 × 10^6^	3.8	3.45 (10.8)	47 (3.9)	73 (4.6)	97.6 (0.3)

^1^*M_n_*–number average molar mass; *M_w_*–weight average molar mass; L/RALS–low-and right-angle light scattering (LALS) and RALS were employed to determine absolute molecular mass; ^2^*M_w_* expected–molar mass declared by producer; $M_{w}=|\left(M_{w}\mathrm{expected}\right)-M_{w}|/\left(M_{w}\mathrm{expected}\right)\times 100\%$; ^3^ intrinsic viscosity; ^4^ *R_η_*–viscosimetric radius; *R_g_*–gyration radius; ^5^ calculated from differential refractometer using dn/dc = 0.129.

**Table 2:** SEC results of starch samples dispersed in aqueous solution of urea (2.3 M)/NaOH (1 M) at 0 °C for 24 h and NaOH or KOH at 65 °C for 1h; the values of the coefficient of variation (%) were given in brackets.

Starch Origin	Dispersion	*M_n_*^1^ (Da)	*M_w_*^1^ (Da)	*M_w_*/*M_n_*^1^	*R_g_*^1^ (nm)	Recovery ^1^ (%)
corn	urea/NaOH	7.85 × 10^6 a^ (3.6)	2.13 × 10^7 b^ (7.4)	2.7 ^b^ (3.8)	122 ^b^ (11.3)	39.0 ^b^ (8.9)
waxy corn	urea/NaOH	4.11 × 10^7 c^ (5.9)	5.27 × 10^7 e^ (2.1)	1.3 ^a^ (8.0)	124 ^b^ (2.1)	28.3 ^a^ (8.9)
potato	urea/NaOH	7.30 × 10^7 d^ (2.2)	9.16 × 10^7 f^ (2.3)	1.3 ^a^ (0.1)	185 ^c^ (1.2)	42.2 ^b^ (5.8)
waxy potato	urea/NaOH	2.54 × 10^8 e^ (3.0)	2.94 × 10^8 g^ (0.5)	1.2 ^a^ (2.6)	252 ^d^ (7.5)	21.2 ^a^ (8.0)
potato amylose	urea/NaOH	3.91 × 10^5 a^ (3.2)	1.37 × 10^6 a^ (1.9)	3.5 ^c^ (1.3)	62 ^a^ (12.3)	99.8 ^d^ (1.5)
corn	0.5 M NaOH	2.57 × 10^7 b^ (6.2)	3.09 × 10^7 c,d^ (5.1)	1.2 ^a^ (1.1)	113 ^b^ (10.2)	38.9 ^b^ (2.1)
corn	1.0 M NaOH	2.93 × 10^7 b,c^ (4.8)	3.45 × 10^7 d^ (4.6)	1.2 ^a^ (0.2)	120 ^b^ (4.7)	45.2 ^b^ (4.0)
corn	2.0 M KOH	2.37 × 10^7 b^ (2.8)	2.89 × 10^7 c^ (1.2)	1.2 ^a^ (1.7)	110 ^b^ (6.6)	57.5 ^c^ (3.7)

^1^*M_n_*–number average molar mass; *M_w_*–weight average molar mass; *M_w_*/*M_n_*–polydispersity index; *R_g_*–gyration radius; recovery was calculated from differential refractometer using dn/dc = 0.129; ^a–g^ different letters show significant differences in means (*p* < 0.05) between values in columns.
